# Acute hormonal response to glucose, lipids and arginine infusion in overweight cats

**DOI:** 10.1017/jns.2014.4

**Published:** 2014-04-30

**Authors:** Lucile J. M. Martin, Thomas A. Lutz, Caroline Daumas, Philippe Bleis, Patrick Nguyen, Vincent Biourge, Henri J. W. Dumon

**Affiliations:** 1 Unité de Nutrition et Endocrinologie, Oniris, LUNAM University, Nantes, France; 2 Institute of Veterinary Physiology, Vetsuisse Faculty University of Zurich, Zurich, Switzerland; 3 Royal Canin, Centre de Recherches, Aimargues, France

**Keywords:** Overweight cats, Nutrient load, Ghrelin, Glucose, Insulin, AG, acylated ghrelin, ARG, arginine, BFM, body fat mass, BW, body weight, ME, metabolisable energy, PRL, prolactin

## Abstract

In cats, the incidence of obesity and diabetes is increasing, and little is known about specific aspects of the endocrine control of food intake in this species. Recent data suggest that ghrelin has an important role in the control of insulin secretion and vice versa, but this role has never been demonstrated in cats. Here we aimed to improve our understanding about the relationship between insulin, amylin and ghrelin secretion in response to a nutrient load in overweight cats. After a 16 h fast, weekly, six overweight male cats underwent randomly one of the four testing sessions: saline, glucose, arginine and TAG. All solutions were isoenergetic and isovolumic, and were injected intravenously as a bolus. Glucose, insulin, acylated ghrelin (AG), amylin and prolactin were assayed in plasma before and 10, 20, 40, 60, 80 and 100 min after the nutrient load. A linear mixed-effects model was used to assess the effect of bolus and time on the parameters. A parenteral bolus of glucose or arginine increased insulin and ghrelin concentrations in cats. Except for with the TAG bolus, no suppression of ghrelin was observed. The absence of AG suppression after the intravenous load of arginine and glucose may suggest: (1) that some nutrients do not promote satiation in overweight cats; or that (2) AG may be involved in non-homeostatic consumption mechanisms. However, the role of ghrelin in food reward remains to be assessed in cats.

The popularity of the cat as a pet has dramatically increased in the last 20 years. This success has been associated with many changes for feline life: neutering, decreased physical activity and indoor confinement. In parallel, the typical diet of pet cats has changed from a carnivorous to an omnivorous regimen since many cats are now fed with very palatable high-energy-dense commercial dry foods. All of these modifications have been recognised to increase food intake and decrease energy expenditure, which may eventually lead to obesity and type 2 diabetes mellitus^(^^1^^)^. Whether high-carbohydrate diets are a risk factor for obesity and type 2 diabetes mellitus remains an unsolved question at the present time^(^^1^^,^^2^^)^.

In cats, the main source for blood glucose is gluconeogenesis from amino acids^(^^3^^)^ and amino acids are strong stimulators of pancreatic hormone release, especially insulin^(^^4^^)^. Although cats are strict carnivores, they appear to be quite efficient at digesting processed starches, even though they may have a limited ability to metabolise some mono- and disaccharides^(^^5^^–^^7^^)^. Cats have minimal activity of hepatic glycogen synthase^(^^8^^)^ and a lower ability than dogs to remove glucose from the bloodstream after a meal^(^^9^^)^.

In mammals, many signalling molecules are involved in the control of food intake, glucose homeostasis and energy expenditure^(^^10^^)^. Insulin is the traditional hormone cited for the control of blood glucose concentration. After a meal, β-cells of the pancreas secrete insulin in response to a rise in blood glucose, but this response is also proportional to body fat^(^^10^^)^. The role of insulin is not limited to blood glucose regulation. Other metabolic effects of insulin include stimulation of the incorporation of amino acids into proteins, and inhibition of the release of NEFA from adipose tissue^(^^11^^)^. Moreover, insulin acts peripherally to increase energy storage, and centrally to reduce food intake and to increase energy expenditure^(^^10^^)^. Similarly to leptin, insulin is considered to be an adiposity signal because it is secreted in proportion to body fat mass (BFM) and influences food intake and body weight (BW)^(^^10^^)^.

Amylin is also considered to be an adiposity factor^(^^10^^)^. This hormone is co-secreted with insulin. It delays gastric emptying and reduces food intake. Amylin has also been shown to increase the anorexic effect of other hormones such as cholecystokinin, insulin and leptin^(^^12^^–^^15^^)^. Insulin and amylin have complementary effects on glucose metabolism and food intake. During the postprandial period, insulin stimulates peripheral glucose uptake, whereas amylin controls glucose appearance into the bloodstream by the control of gastric emptying, suppression of postprandial glucagon secretion and by inducing satiation^(^^15^^)^. In conditions associated with insulin resistance such as obesity, amylin levels are often increased in parallel with insulin. It has been shown that chronic exposure to amylin decreases food intake and BW gain^(^^16^^)^; further, even in chronic hyperamylinaemia amylin signalling is not down-regulated, and amylin is able to suppress food intake^(^^16^^)^.

Presently, only one peripherally released orexigenic hormone has been discovered: ghrelin. Ghrelin is mainly but not only secreted from the stomach, and circulating levels are higher during fasting. Two major forms have been recognised^(^^17^^)^: acylated ghrelin (AG) and deacylated ghrelin. Both forms control insulin secretion and glucose metabolism. AG has traditionally been considered to be a hunger signal, being sensed in the central nervous system; accordingly, exogenous ghrelin has been reported to stimulate food intake, BW gain and adiposity when administered peripherally or centrally to rodents^(^^18^^)^.

Ghrelin also stimulates prolactin (PRL) release^(^^19^^)^. PRL is involved in the control of food intake, especially but not exclusively during periods of reproduction^(^^20^^)^. In rats, PRL receptors are expressed on oxytocin neurons in the paraventricular hypothalamus^(^^21^^)^, neurons implicated in food intake and energy metabolism. PRL receptor activation is also involved in the development of islet cells of the pancreas and in the increase in islet cell mass during pregnancy. In brown adipose tissue, the effects of PRL are numerous^(^^22^^)^: PRL stimulates proinsulin gene expression, and, in the presence of insulin, increases Ob (leptin) gene expression as well as leptin release.

In a recent study, we provided the first results on postprandial AG concentrations in cats and underlined several discrepancies between cats and previous reports in human subjects and rodents^(^^23^^)^. In that study, we compared the effect of three meals with different protein:fat ratios on the postprandial concentrations of insulin, AG and amylin in lean and obese cats. We showed that food intake did not differ among diets, but, interestingly, obese cats consumed significantly less food than lean cats. Obesity induced significantly higher postprandial responses of blood glucose and ghrelin, but had no effect on insulin and amylin variations. The main effect on the different parameters was related to the protein content of the diet. We unexpectedly observed that AG concentrations rose significantly after a meal in cats. Nevertheless, with regard to the total amount of food spontaneously consumed with the three diets, the highest postprandial AG was not associated with higher food intake.

As very few studies have been conducted in cats, further investigation was required for a better understanding of the effect of ghrelin. In our previous study, we questioned why AG was not suppressed after meal ingestion. We therefore decided to use each nutrient separately to depict the time course of AG concentration after an intravenous load. The relationship between insulin, amylin and ghrelin secretion and their relevant roles in glucose metabolism in overweight cats were considered here. We hypothesised that: (1) AG concentration would promptly decrease in cats after a nutrient load (glucose, arginine (ARG) or NEFA); and (2) there was an inverse relationship between AG and insulin concentrations.

## Materials and methods

### Animals

A group of six overweight neutered domestic short hair adult male cats from different genetic backgrounds (body condition score 7 on a nine-point scale) were included in the study. Mean BW was 6·2 (sd 0·5) kg and mean age was 6·9 (sd 1·8) years. Before the start of the study, the cats were group housed and fed free choice for at least 2 months the same balanced maintenance diet (Neutered Cat, Young Adult; Royal Canin).

During the study, the cats were weighed weekly and body condition score was noted. A clinical and biochemistry examination was performed before the cats were included in the study.

### Protocol

The experimental protocol was reviewed and approved by the Royal Canin Committee for Animal Ethics and Welfare. Husbandry and use of the cats were in accordance with current French and international regulations concerning animals used for experiments (authorisation no. D 00 66 894). Particular attention was paid to the guidelines designed to promoting well-being of the cats in a safe, enriched environment. Except during periods of handling during the study, the cats were kept together in a large cattery with inside and outside areas. Room lighting consisted of 12 h light and dark periods from 06.00 to 18.00 hours and the inside temperature ranged from 18°C to 21°C.

### Methods

After the period of adaptation on the same balanced cat food, cats were randomly assigned successively to one of the four intravenous solutions tested: saline, ARG, fatty acids or glucose.

The experimental procedures were chosen to limit stress and to promote safe handling of the cats. The day before each test, cats were anaesthetised using the following protocol: atropine sulfate (0·05 mg/kg subcutaneously) and, 15 min later, a mixture of tiletamine/zolazepam (10 mg/kg intramuscularly). A 6 cm sterile catheter (Leader-Cath; Vygon) was then inserted in the jugular vein using the Seldinger method and sutured to the skin (3/0 Prolene; Ethicon) to allow painless blood sampling. Catheter patency was maintained by flushing with a heparinised (50 IU/ml) physiological saline solution. A head cone was placed to prevent the catheter from being torn off during the night.

After a 16 h fast, all cats underwent one of the following four testing sessions in a random order: (1) saline; (2) d-glucose (G30; B. Braun Medical SA); (3) ARG (l-arginine hydrochloride 21 %; B. Braun Melsungen AG); and (4) TAG from soya oil (Intralipid 20 %; Fresenius Kabi). The cats underwent one test per week for 4 weeks. All solutions were injected intravenously as a bolus via a separate catheter placed in the cephalic vein. For all the tests, the amount injected represented 4 % of daily maintenance metabolic energy requirement for an adult overweight cat according to the National Research Council^(^^24^^)^ (544 × BW^0·4^ kJ (130 × BW^0·4^ kcal) metabolisable energy (ME)/d) (isoenergetic tests). The total volume injected was adjusted to be identical for each test (isovolumic tests). As the largest calculated volume was for l-arginine, all the other volumes were adjusted on its basis to provide isoenergetic loads. For example, for a 6 kg cat, the volume of each solution was 8·3 ml, 11·9 ml and 5·5 ml for d-glucose, ARG and TAG, respectively. All the volumes were adjusted to 11·9 ml for all the solutions, including saline.

As it has been shown that oral infusion of glucose can decrease the plasma concentration of ghrelin as soon as 30 min after ingestion in human subjects and in rats^(^^25^^)^, blood samples were taken before (10 min before each bolus = baseline) and 10, 20, 40, 60, 80 and 100 min after injection to depict the acute effect of the nutrient load on glucose, insulin, AG, amylin and PRL concentrations. All blood samples were handled similarly: whole blood (2 ml) was collected through the jugular catheter and placed in tubes containing EDTA + aprotinin (0·6 trypsin inhibitor units/ml of whole blood, RK-APRO; Phoenix Pharmaceuticals Inc.) for plasma collection. Immediately after collection, the tubes were preserved on ice (at about 0–4°C) until centrifugation (< 30 min). The tubes were centrifuged (Sigma 2 K 15; Sigma Laborzentrifugen GmbH) at 3000 ***g*** for 10 min at 4°C. The plasma for hormone measurements was stored at < −80°C pending analysis.

### Analyses

Body composition was determined from isotope dilution of ^2^H_2_O at the start of the study^(^^23^^)^. For hormone analyses, commercially available kits were used. All assays had previously been validated for use in cats; the assay procedures were performed according to the manufacturers' instructions. Insulin was assayed in duplicate using the porcine insulin RIA kit (catalogue no. PI-12 K-85 K; Linco Research, Inc.)^(^^23^^)^. Amylin was assayed in duplicate with a RIA kit specifically developed for cats (catalogue no. RK-017-01; Phoenix Pharmaceuticals, Inc.)^(^^23^^)^. Plasma ghrelin levels were measured in duplicate with a human active ghrelin RIA kit from Linco Research (catalogue no. GHRA-88HK) that shows significant cross-reactivity with feline ghrelin^(^^23^^)^. PRL was assayed in triplicate as previously described in cats^(^^26^^)^.

### Statistical analysis

Considering the large variation in baseline data (Table 1), the results were calculated as change from baseline for each respective cat. A linear mixed-effects model was used to assess the effect of body composition (df = 1), bolus (df = 3) and time (df = 4) on the measured parameters. The fixed effects were the type of bolus (glucose, ARG, TAG or saline) and time, and the random effect corresponded to individuals (cats). The normality of the residuals was checked for each model to assess the validity of the test.

**Table 1.** Baseline concentrations for glycaemia, insulin, amylin and acylated ghrelin* (Mean, minimum and maximum values and standard errors)

	Cats × testing sessions (*n*)	Mean	Minimum	Maximum	sem
Blood glucose (mmol/l)	6 × 4	5·2	4·2	7·6	0·15
Insulin (pmol/l)	6 × 4	196·0	55·6	444·5	19·4
Amylin (pg/ml)	6 × 4	50·5	27·0	92·4	3·4
Acylated ghrelin (pg/ml)	6 × 4	43·4	26·4	79·4	3·0
Prolactin (ng/ml)	6 × 4	40·3	15·4	93·6	3·8

* Baseline concentrations were assayed in plasma samples before the administration of each bolus. Mean baseline concentrations did not differ between cats.

When appropriate (*P* < 0·05), the linear mixed-effects model was followed by a Tukey's *post hoc* test to assess the effect of bolus, time or BFM on the measured parameters. Pearson's correlation tests were also used to measure the strength of association among data. Data were expressed as mean values with their standard errors, and *P* < 0·05 was considered significant. All statistical analyses were performed with XLstat-Pro 2011 software (Addinsoft SARL).

## Results

All cats remained healthy during the study on the basis of a weekly physical examination, the lack of any clinical signs and biochemistry analysis. Mean BFM was 34·5 (sd 3·3) %.

### Baseline concentrations

Baseline concentrations are presented in Table 1. Mean baseline concentrations of insulin, amylin, PRL and ghrelin did not differ between cats. BFM and blood glucose were positively correlated (*P* = 0·008) (data not shown). BFM was also significantly negatively correlated with mean PRL concentrations (BFM < 35 %: mean concentration = 50·8 ng/ml; BFM > 35 %: mean concentration = 24·7 ng/ml; *P* = 0·001) (data not shown).

### Blood glucose

**Fig. 1.**
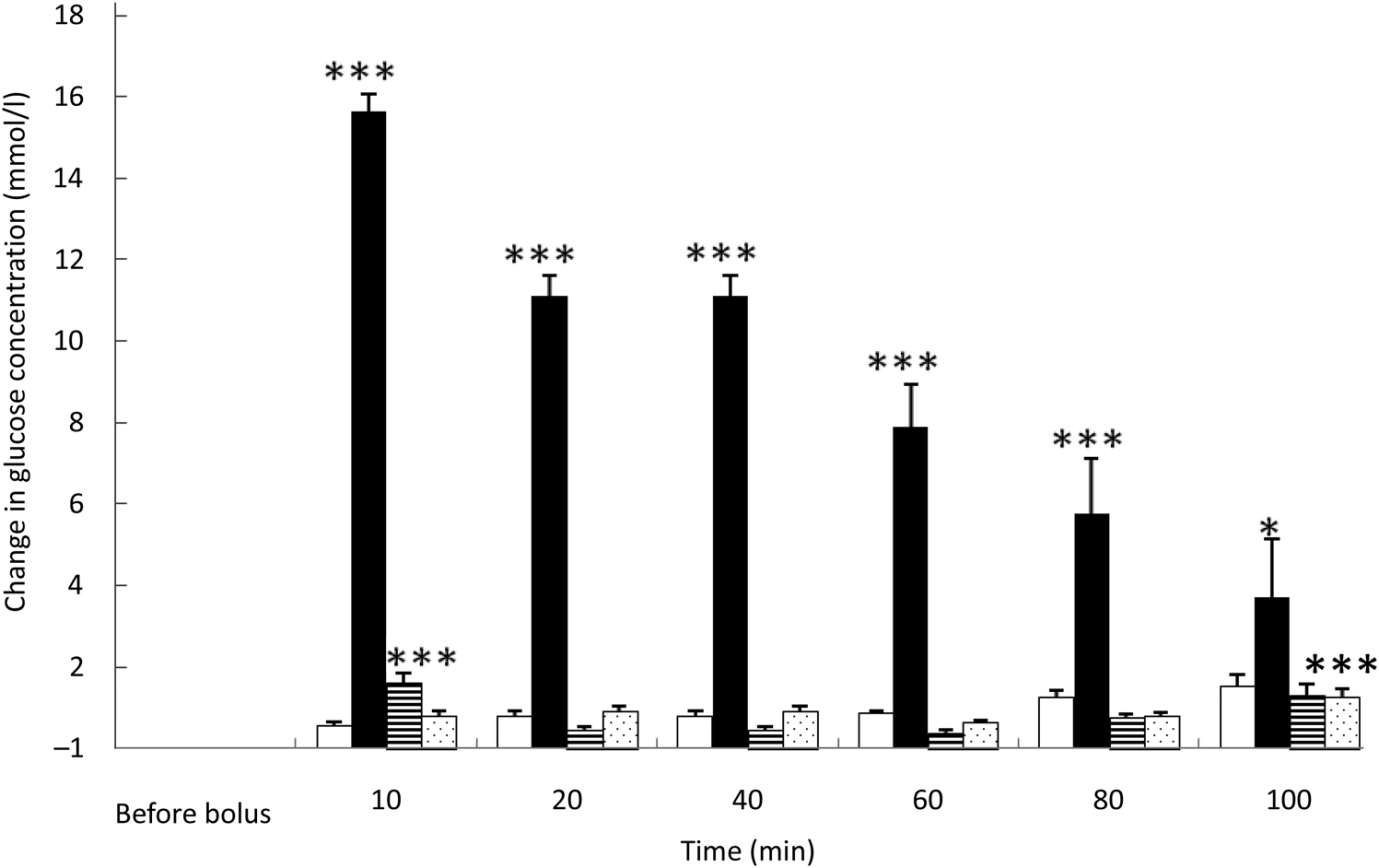
Glucose variations from baseline after saline (□), glucose (■), arginine (≡) and TAG (░) intravenous loads. Values are means, with standard errors represented by vertical bars. There was no significant variation in concentrations within the test after the saline bolus. The concentration of glucose was significantly increased from baseline 10 min (*P* < 0·0001), 20 min (*P* < 0·0001), 40 min (*P* < 0·0001), 60 min (*P* < 0·0001), 80 min (*P* = 0·0001) and 100 min (*P* = 0·021) after intravenous glucose injection. The concentration of glucose was significantly higher than baseline 10 min (*P* < 0·0001) after the arginine bolus injection. The concentration of glucose was significantly higher than baseline 100 min (*P* < 0·0001) after the TAG bolus injection. Mean value was significantly different from that at baseline: *P < 0·05, ***P < 0·001.

Saline administration did not significantly modify blood glucose level (Fig. 1).

As expected, glycaemia was the highest after the glucose bolus (*P* < 0·0001) (Fig. 1), showing the main increase 10 min after injection (+15·1 (sem 1·5) mmol/l; *P* < 0·001), then slowly decreasing over time. After 100 min, blood glucose had not returned to its baseline concentration (*P* = 0·021). Despite the low energy load of the intravenous infusion (4 % of daily maintenance metabolic energy requirement), the amplitude of glycaemic excursions was higher than after a meal for cats^(^^23^^)^.

Overall glycaemia was increased after ARG administration (*P* < 0·0001) (Fig. 1). There was a small, transient but significant rise of glycaemia from baseline10 min after the ARG bolus (*P* < 0·001), then a rapid decrease to baseline: as soon as 20 min after injection, glycaemia returned to baseline (*P* = 0·785). The observed variations in blood glucose were small and remained similar to postprandial concentrations.

After TAG injection, in general, blood glucose varied significantly from baseline (*P* = 0·001). Blood glucose concentration was higher than baseline 100 min after TAG injection (Fig. 1).

### Insulin concentration

**Fig. 2.**
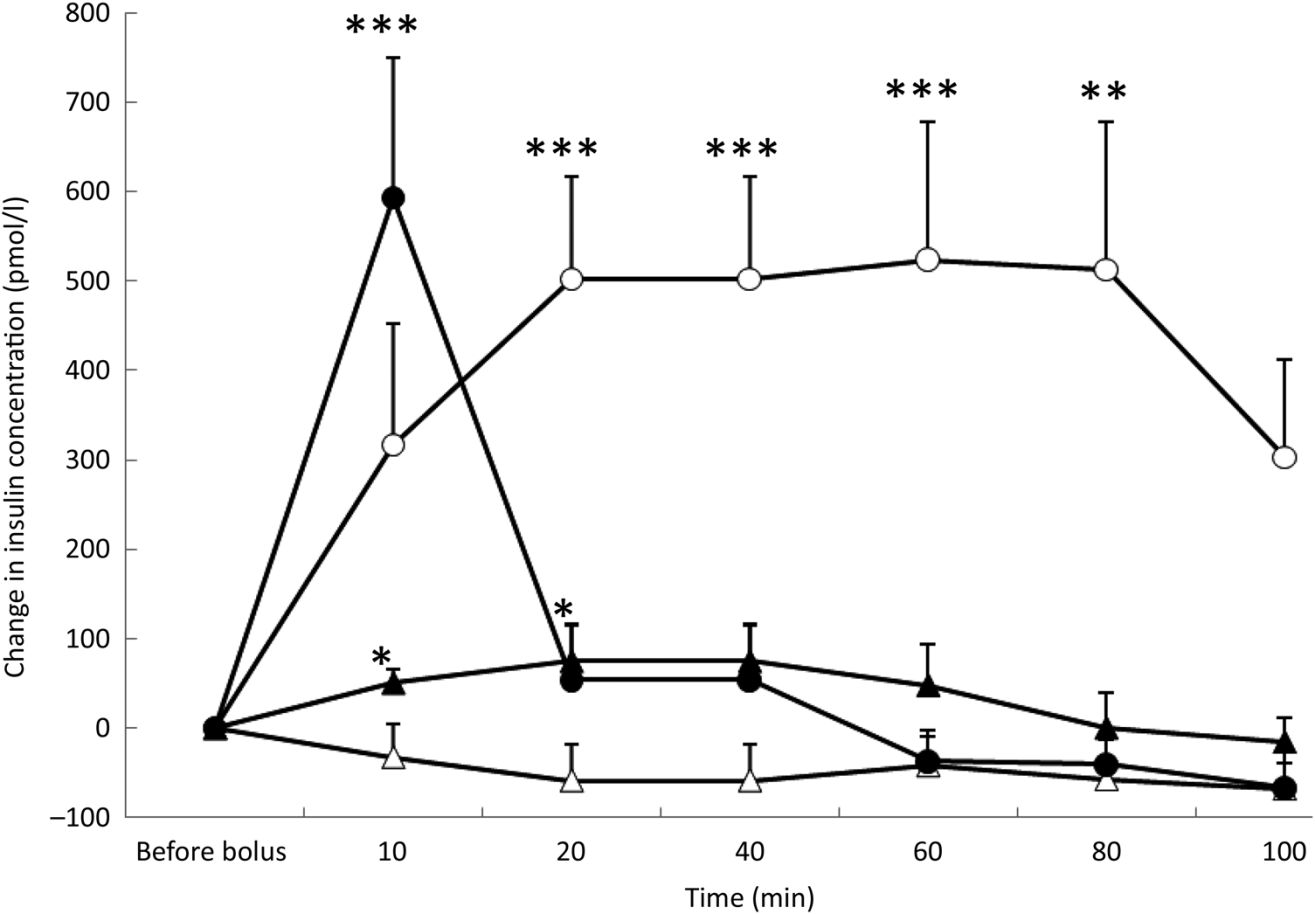
Insulin variations from baseline after saline (△), glucose (○), arginine (•) and TAG (▴) intravenous loads. Values are means, with standard errors represented by vertical bars. There was no significant variation in concentrations within the test after the saline bolus. The concentration of insulin was significantly increased from baseline 20 min (*P* = 0·004), 40 min (*P* = 0·004), 60 min (*P* = 0·003) and 80 min (*P* = 0·003) after glucose administration. Insulin was significantly increased from baseline 10 min (*P* < 0·0001) after the arginine bolus injection. Insulin concentration increased significantly from baseline at 10 min (*P* = 0·027) and 20 min (*P* = 0·027) after the TAG bolus injection. Mean value was significantly different from that at baseline: *P < 0·05, **P < 0·01, ***P < 0·001.

The saline bolus did not significantly affect the concentrations of insulin during the 100 min following its administration (Fig. 2).

After the glucose bolus and in response to the significant increase in glycaemia, there was a significant increase in insulin concentrations (*P* = 0·032) which remained high throughout the study period (Fig. 2). After 100 min, the mean insulin variation was  + 313 pmol/l but was not significantly different from baseline (*P* = 0·074).

At 10 min after ARG injection, insulin concentration was significantly increased from baseline (*P* < 0·0001), and then it decreased promptly to reach baseline levels at 20 min (Fig. 2).

TAG induced a significant prompt but transient increase in insulin (*P* = 0·033), peaking at 20 min (*P* = 0·027) and at 40 min (*P* = 0·027), and then insulin concentration returned to baseline (Fig. 2). With the TAG bolus, time course of insulin concentration was influenced by the BFM of the cats (*P* = 0·0001) because cats with a BFM < 35 % showed the highest mean increase ( + 83·3 pmol/l *v.* −19·4 pmol/l for cats with a BFM > 35 %) (data not shown).

### Acylated ghrelin concentrations

**Fig. 3.**
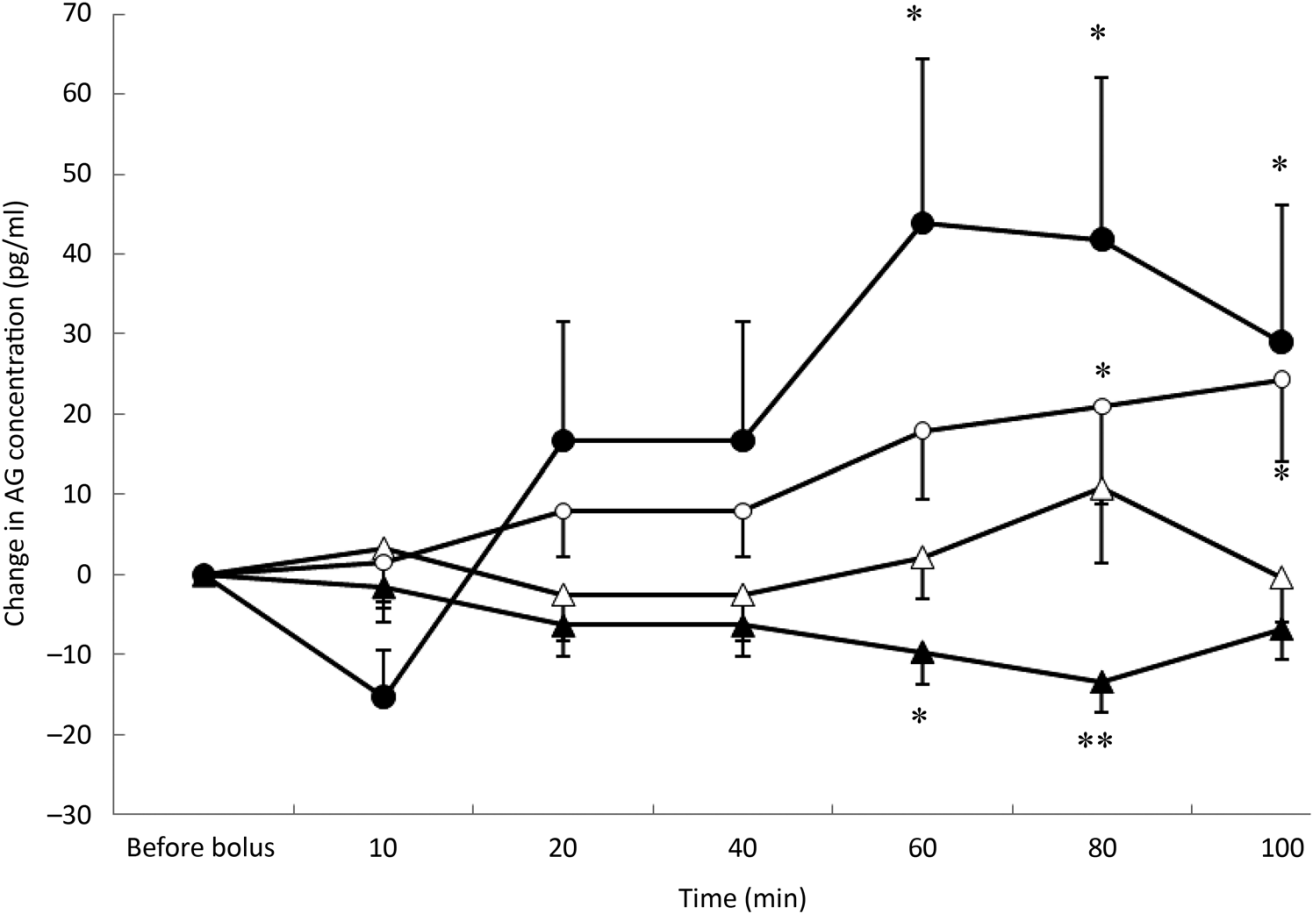
Acylated ghrelin (AG) variations from baseline after saline (△), glucose (○), arginine (•) and TAG (▴) intravenous loads. Values are means, with standard errors represented by vertical bars. There was no significant variation in concentrations within the test after the saline bolus. The concentration of AG was significantly increased from baseline 80 min (*P* = 0·025) and 100 min (*P* = 0·023) after glucose administration. AG significantly increased from baseline at 60 min (*P* = 0·001), 80 min (*P* = 0·002) and 100 min (*P* = 0·027) after arginine administration. AG was significantly lower than baseline at 60 min (*P* = 0·013) and 80 min (*P* = 0·001) after the TAG bolus injection. Mean value was significantly different from that at baseline: *P < 0·05, **P < 0·01.

Saline did not significantly affect the concentrations of AG during the 100 min following its administration (Fig. 3).

Plasma AG significantly increased from baseline over time (*P* = 0·05) (Fig. 3) after glucose. The concentration of AG was significantly increased at 80 min (*P* = 0·025) and 100 min (*P* = 0·023).

The acute administration of ARG was followed by a significant increase in AG concentrations from baseline (*P* < 0·0001) that was preceded by a transient but non-significant reduction in AG concentration at 10 min (Fig. 3). The concentrations of AG were significantly higher than baseline at 60 min (*P* = 0·001), 80 min (*P* = 0·002) and 100 min (*P* = 0·027) and did not return to baseline values during the duration of the study.

After TAG administration, there was a significant progressive decrease in AG concentrations (*P* = 0·021) (Fig. 3). AG concentrations were significantly lower than baseline at 60 min (*P* = 0·013) and 80 min (*P* = 0·001). Such as for insulin concentrations, time course of AG was modified according to the BFM of the cats (*P* = 0·002); cats with a BFM > 35 % showed the highest mean decrease (−11·7 pg/ml *v.* −0·9 pg/ml for cats with a BFM < 35 %) (data not shown).

### Amylin concentrations

**Fig. 4.**
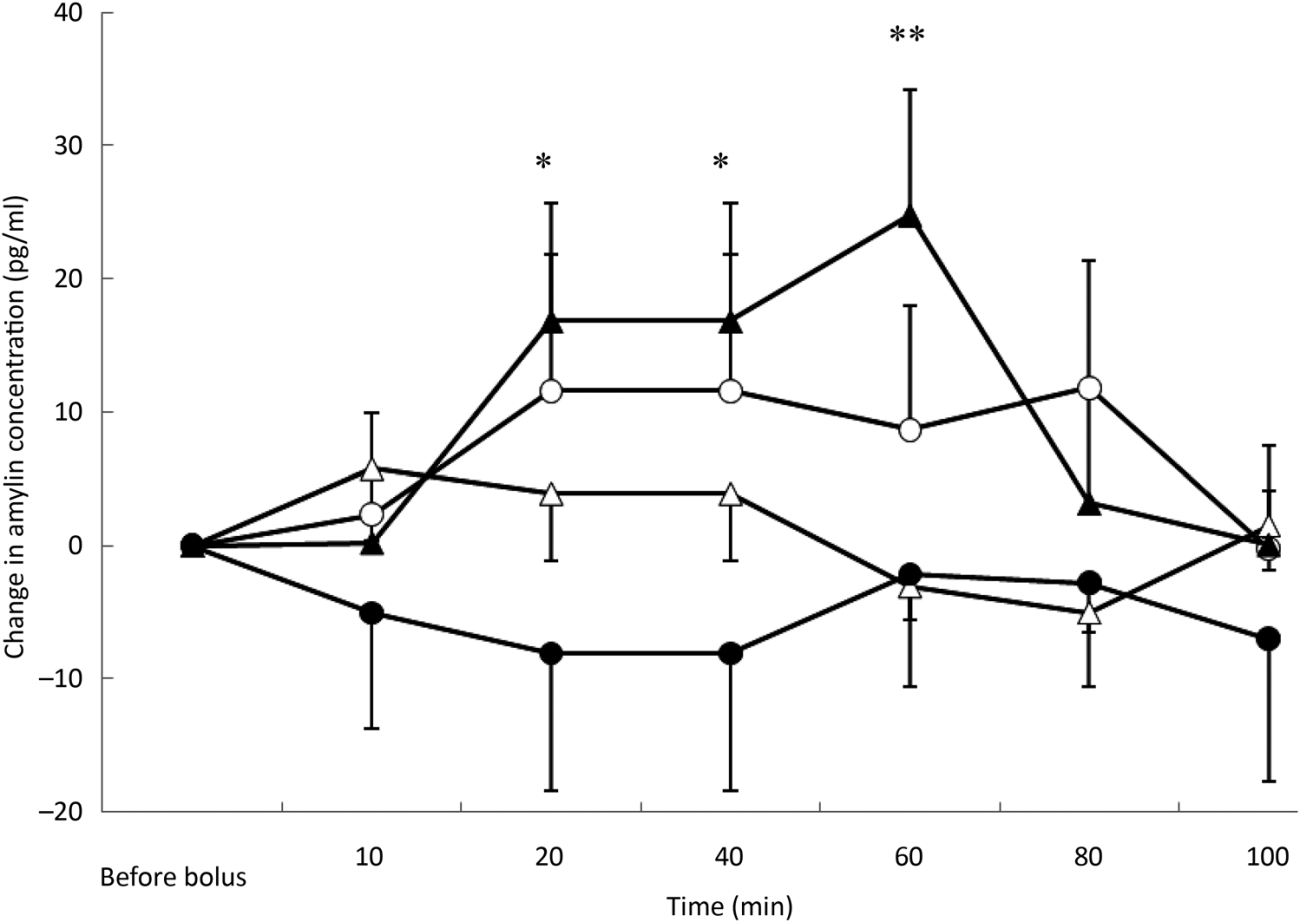
Amylin variations from baseline after saline (△), glucose (○), arginine (•) and TAG (▴) intravenous loads. Values are means, with standard errors represented by vertical bars. There was no significant variation in amylin concentration during the saline, glucose and arginine tests. Amylin variations were significantly higher than baseline after the TAG bolus at 20 min (*P* = 0·027), 40 min (*P* = 0·027) and 60 min (*P* = 0·002). Mean value was significantly different from that at baseline: *P < 0·05, **P < 0·01.

Amylin concentrations were affected by both the type of bolus (*P* = 0·003) (Fig. 4) and the BFM of the cats (*P* = 0·048). There was a significant increase in amylin concentration at 20 min (*P* = 0·027), 40 min (*P* = 0·027) and 60 min (*P* = 0·002) after the TAG bolus. Amylin concentrations were the highest for cats with a BFM < 35 % (mean variation +7·1 pg/ml) whereas cats with a BFM > 35 % showed the lowest variations (mean variation −0·8 pg/ml) (data not shown). However, there was no significant variation in amylin concentration with time for the saline, ARG and glucose tests, despite significant increases in insulin concentrations after glucose and ARG administration (Fig. 4).

### Prolactin concentrations

**Fig. 5.**
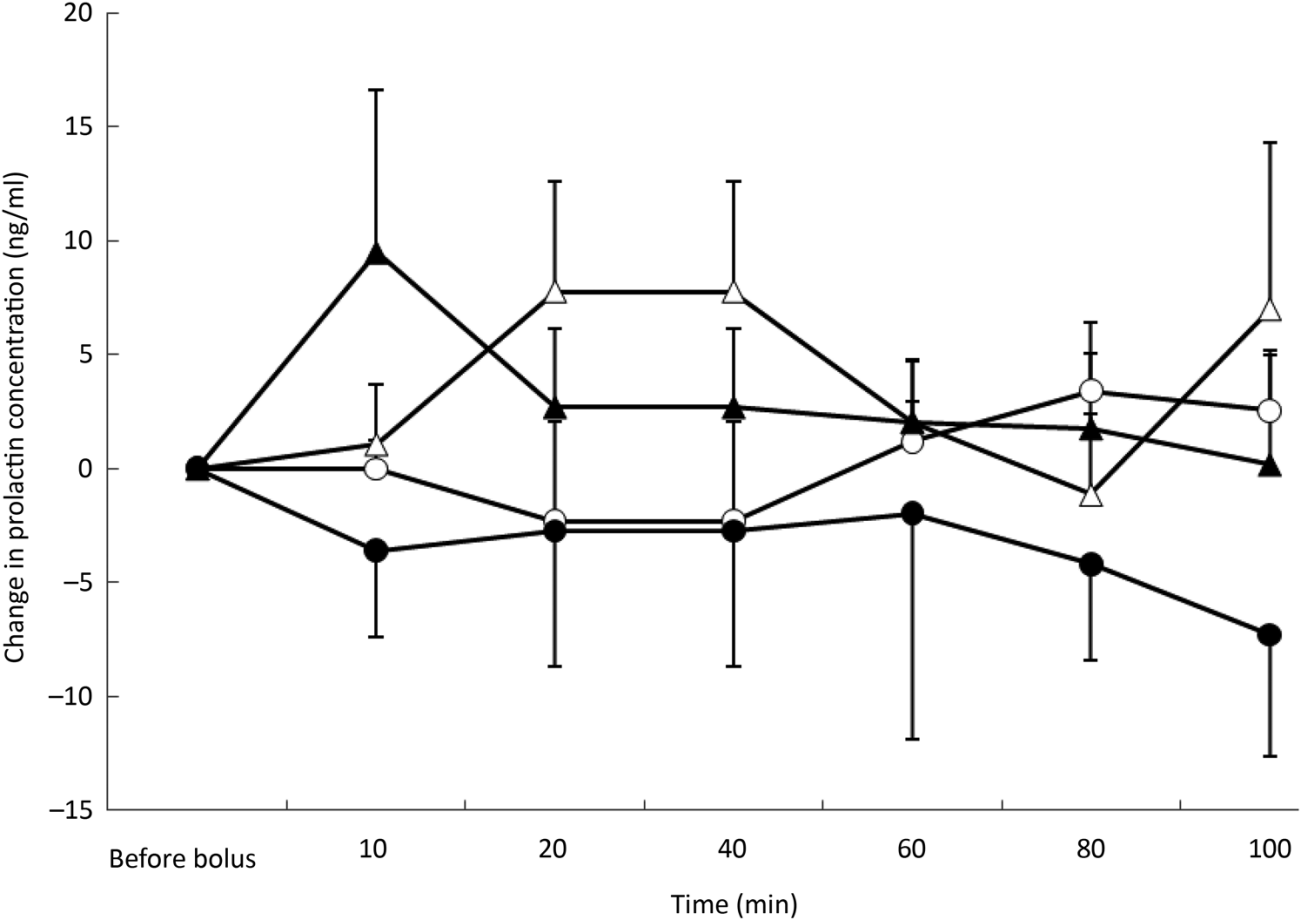
Prolactin variations from baseline after saline (△), glucose (○), arginine (•) and TAG (▴) intravenous loads. Values are means, with standard errors represented by vertical bars. There was no significant variation from baseline with time but overall concentrations were the lowest after the arginine load (*P* = 0·002).

Post-test PRL concentrations were affected by the type of nutrient (*P* = 0·002) but not by BFM or time. Mean concentrations were lowest during the ARG test (mean variation −6·5 ng/ml) whereas mean variations were not different among the saline (mean variation −0·2 ng/ml), glucose (mean variation 0·2 ng/ml) and TAG (mean variation 2·2 ng/ml) tests (Fig. 5).

### Pearson's correlations among data

When pooling all data, the Pearson's correlation test showed a significant relationship between glucose and insulin (*P* < 0·0001; *r* 0·48) and between amylin and insulin (*P* = 0·01; *r* 0·21). PRL was correlated with the following parameters: amylin (*P* = 0·034; *r* 0·16); insulin (*P* = 0·029; *r* 0·17); and glycaemia (*P* = 0·0008; *r* 0·25). Nevertheless, except for insulin and glucose, the correlations were weak.

### AUC

AUC were calculated for glucose, insulin, amylin and ghrelin (Table 2) from baseline concentrations.

**Table 2.** AUC for blood glucose, insulin, amylin and acylated ghrelin* (Mean values and standard deviations)

	Mean	sd	Test effect (*P*)
AUC glucose (mmol/l × min)			0·012
Saline	43·5^a^	24·6	
Glucose test	838·5^b^	165·6	
Arginine test	34·1^a^	13·4	
TAG test	30·1^a^	16·7	
AUC insulin (pmol/l × min)			0·002
Saline	1037^a^	1952	
Glucose test	38008^d^	15860	
Arginine test	14173^c^	7522	
TAG test	6618^b^	5809	
AUC amylin (pg/ml × min)			NS
Saline	296·0	275·3	
Glucose test	955·7	1269·1	
Arginine test	650·4	808·8	
TAG test	1690·5	1773·3	
AUC acylated ghrelin (pg/ml × min)			NS
Saline	671·8	645·9	
Glucose test	1435·0	1304·8	
Arginine test	2876·3	1784·6	
TAG test	96·5	96·0	

* The AUC for glucose was significantly higher after the glucose bolus and did not differ between the saline, arginine and TAG tests. The AUC for insulin differed significantly between the four tests and was the highest after the glucose bolus. There was no statistical difference for the AUC for amylin and the AUC for ghrelin.

^a,b,c,d^ Mean values within an outcome with unlike superscript letters were significantly different (*P* < 0·05).

The AUC for glucose was significantly higher after glucose injection and did not differ between the saline, ARG and TAG tests (*P* = 0·012). The AUC for insulin differed significantly between the four tests and was the highest after glucose administration (saline 1037 (sem 1952) pmol/l × min < TAG 6618 (sem 5809) pmol/l × min < ARG 14173 (sem 7522) pmol/l × min < glucose 38008 (sem 15860) pmol/l × min; *P* = 0·002). There was no statistical difference for the AUC for amylin and the AUC for ghrelin due to great variations in concentrations among cats.

## Discussion

The present study was intended to examine whether nutrients affect variations in glucose, insulin, amylin, AG and PRL in overweight cats, all these hormones being signals for the control of glucose metabolism, food intake and BW. To our knowledge, this is the first time that the relationship between the intravenous load of a single nutrient and endocrine factors involved in metabolism and appetite control has been investigated in cats. The present results showed that responses were influenced by the type of nutrient.

In human subjects and rodents, studies examining the effect of a single nutrient on ghrelin secretion are conflicting. Ghrelin secretion is affected by the class of nutrient, but it is not clear which factors are directly involved in the control of its secretion. In humans and rats, Gil-Campos *et al.*^(^^18^^)^ found that glucose and amino acids suppressed ghrelin secretion more rapidly and strongly than lipids. However, conversely, in normal human subjects, Broglio *et al.*^(^^27^^)^ showed no ghrelin suppression after an ARG or NEFA load. In the present study, no suppression but, rather surprisingly, an increase was observed after the glucose or the ARG tests in cats. The TAG bolus induced a slow AG decrease from baseline for 80 min then an increase to reach baseline again.

In our cats, the AG response might be related to the magnitude of the glycaemic response: there was a notable rise in AG concentration after the glucose or ARG boluses, without any significant suppression of its concentrations. After TAG injection, the small variations of glycaemia from baseline were accompanied by a slow decline in AG concentrations. A strong positive association was previously demonstrated between 24 h mean peripheral concentrations of AG and postprandial glucose in human subjects^(^^28^^)^ and the present results suggest a similar mechanism in cats.

Interestingly, about 10 years ago, Tschöp *et al.*^(^^29^^)^ demonstrated that ghrelin induces a switch from lipid to carbohydrate as the fuel for energy production. Using indirect calorimetry, they showed that a single subcutaneous injection of ghrelin significantly increased the respiratory quotient of the rats, indicating a higher carbohydrate and a reduced fat metabolic utilisation, without any changes in energy expenditure and locomotor activity. This specific ghrelin effect might be independent from growth hormone or neuropeptide Y release. If such a mechanism existed in cats, it could be supposed that the rise in AG concentration after glucose or ARG injection might be a physiological response to promote the metabolic utilisation of glucose as the fuel for energy production.

Nevertheless, when insulin and AG variations were considered together, a glucose bolus induced an increase in both hormones. Conversely, an ARG or TAG bolus induced an inverse relationship in insulin and AG variations: first an increase in insulin and a decrease in AG – notably, the decrease was non-significant after the ARG load – and then when insulin decreased to baseline, an increase in AG was observed.

An inverse relationship between ghrelin and insulin has been previously observed in healthy human subjects^(^^27^^)^. In this study, authors also suggested: (1) the ability of insulin to suppress circulating ghrelin independently from changes in glycaemia; and (2) the ability of ghrelin to decrease insulin concentration and to increase blood glucose by different pathways. In addition, ghrelin is known to play a role in insulin sensitivity^(^^17^^)^. Hence, these data indicate that AG may be a strong regulator of plasma insulin and glucose level in healthy humans^(^^30^^,^^31^^)^ and the results presented here support the idea that a similar mechanism of regulation of blood glucose in cats might be instrumental.

In a previous study, we measured the variations of insulin and AG after the intake of different commercial diets^(^^23^^)^. We used a high-protein/medium-fat diet, a high-fat diet and a balanced diet. During the test meal (which corresponded to 50 % of the daily ration (35 g)), the cats ingested 16·1 g proteins, 4·2 g fats and 6·0 g starch with the high-protein/medium-fat diet, 10·5 g proteins, 7·7 g fats and 8·5 g starch with the high-fat diet and 9·5 g proteins, 4·6 g fats and 11·6 g starch with the balanced diet. In this study, no suppression of AG was observed after the meal and the magnitude of insulin variations in the postprandial state for all three diets was lower than in the present study.

The results were revisited to highlight a possible relationship between insulin and ghrelin in the postprandial period in overweight cats. For the high-fat diet (46 % ME from fat), the response to the test meal also showed an inverse relationship between insulin and AG. A small transient increase in insulin concentration was observed immediately after the test meal (*P* = 0·006) but insulin concentration returned to baseline as soon as 30 min after the meal. The decrease in insulin concentration coincided with an increase in AG concentrations and AG concentrations increased to values higher than baseline 60 min after meal ingestion (*P* = 0·023). For the balanced diet (35 % ME from carbohydrates), no clear inverse relationship appeared, probably due to the balance of proteins, starch and fat in the diet. Nevertheless, AG concentrations were significantly increased 60 min (*P* = 0·038) and 100 min (*P* = 0·033) after meal ingestion, suggesting that cats might not obtain a satisfactory level of satiation after such meals. However, satiation is a complex process and many other factors are involved in this physiological pathway.

With the ‘high-protein/medium-fat’ diet (48 % ME from proteins and 28 % ME from fat), we observed an inverse relationship between insulin and AG, thus depicting a very similar response to the present ARG test^(^^23^^)^. Nevertheless, the concentration of AG did not vary significantly with time from baseline in the previous study. The response to the test meal was probably the consequence of the high level of proteins in the diet. In a recent study, Wei *et al.*^(^^32^^)^ compared the effect of a high-protein diet (47 % ME from protein) and a balanced diet (27 % ME from protein) on energy balance in *ad libitum*-fed obese cats. They found no decrease in food intake in response to the high-protein diet and an increase in energy intake. This result supports our previous observation on AG response after a meal showing no clear suppression of the hormonal concentrations during the postprandial state. Another study also reported that high-protein diets do not induce a decrease in energy intake in cats^(^^33^^)^. This result seems to contradict the statement that protein and amino acids are involved in satiation signalling in cats. In human subjects and in rodents, dietary proteins have a clear influence on the release of the main anorexigenic gut peptides^(^^34^^,^^35^^)^ and, likewise, act to reduce food intake. In human subjects, Long *et al.*^(^^36^^)^ demonstrated that the satiating efficiency of proteins was related to the usual level of protein intake in the diet. They found that the magnitude of satiation decline was related to a higher capacity to oxidise amino acids. In opposition, Russell *et al.*^(^^33^^)^ compared protein oxidation in cats fed a medium-protein diet (35 % ME from protein; 62 % ME from fat) or a high-protein diet (52 % ME from protein; 45 % ME from fat). When the amount of dietary protein increased, protein oxidation also increased significantly while fat oxidation decreased. In their study, the amount of food intake was higher with the high-protein diet (230 g/d) than with the control food (190 g/d) despite similar energy intake.

To understand the mechanisms of food overconsumption in humans, many studies have focused on ghrelin as an interesting target in obesity and other eating disorders. Some studies have shown that central ghrelin action stimulates the intake of high-energy-dense palatable foods, and may play a key role in the choice of highly palatable foods^(^^37^^–^^39^^)^. In addition, ghrelin administration stimulates food-seeking behaviour in both human subjects and rodents^(^^40^^)^. Subsequently, it has been suggested that ghrelin regulates the extra-homeostatic aspects of eating^(^^41^^,^^42^^)^. The extra-homeostatic factors are increased with food palatability and energy content, and may result in overconsumption of food and then in obesity^(^^42^^)^. Ghrelin is now recognised as a key peptide in hedonic signalling of food and possibly in the concept of food addiction^(^^39^^–^^42^^)^. In a recent series of experiments, Dickson and her group^(^^42^^)^ used a rat model to assess the role of ghrelin in food reward. The specific role of endogenous ghrelin in food reward was confirmed by showing that the injection of a ghrelin receptor antagonist decreased the motivation of rats to obtain sucrose pellets. They showed an impact of chronic central ghrelin treatment on the gene expression of dopaminergic and cholinergic receptors in key reward nodes of the central nervous system. Hence, they underlined the potential role of ghrelin as a therapeutic target to suppress overeating of high-energy-dense foods in human subjects^(^^42^^)^. In the present study, the absence of AG suppression after an intravenous load of ARG or glucose may suggest that some nutrients unpredictably might not promote satiation in overweight cats, but the role of ghrelin in food reward has never been documented in this species.

Despite its key role in glucose homeostasis^(^^15^^,^^16^^)^, surprisingly no variation of amylin was observed after the glucose or ARG load. However, after the TAG load, there was an increase in amylin concentration, showing similar variations to insulin. In human subjects with impaired glucose tolerance, amylin release after a glucose load was decreased^(^^43^^)^. It has also been suggested that an altered ratio between amylin and insulin secretion encourages abnormal feeding behaviour under some conditions, such as type 1 diabetes or later stages of type 2 diabetes mellitus^(^^16^^)^; if such a mechanism were relevant in overweight cats, the discrepancy observed between amylin and insulin secretion may be one of the factors involved in the dysregulation of satiation and in BW increase in overweight cats.

As ghrelin modulates lactotroph secretion in human subjects and animals^(^^44^^,^^45^^)^, we hoped to demonstrate a relationship between AG and PRL secretion. Nevertheless, in the present study, we failed to observe such a relationship after a nutrient load.

Some limitations appeared in the present study. We used a small number of cats with large inter-individual variations. Nevertheless, as all the cats underwent all the testing sessions, this increased the statistical power of the study due to intra-individual comparisons. Another drawback was that we did not compare the endocrine response in normal-weight and overweight cats, and responses would probably be different as we previously noted^(^^23^^)^ after a meal. The description of changes in behaviour associated with the nutrient load would be of great interest but we did not have a specific test to validate our observations; hence further studies are needed to associate clinical behaviour with laboratory work.

In conclusion, the present study showed that an intravenous bolus of glucose or ARG increases glucose, insulin and ghrelin responses in overweight cats. The results suggest a significant role for ghrelin in the control of glucose homeostasis in this species, as in humans or rodents. Except for with the TAG bolus, no suppression of ghrelin was observed during the 100 min of the test. The absence of AG suppression after an intravenous load of ARG or glucose suggests: (1) that some nutrients unexpectedly may not promote satiation in overweight cats; and (2) a possible role for ghrelin in food-reward mechanisms in the feline species. However, the role of ghrelin in food reward remains to be assessed in cats.
